# Meaning in life: bidirectional relationship with depression, anxiety, and loneliness in a longitudinal cohort of older primary care patients with multimorbidity

**DOI:** 10.1186/s12877-025-05762-7

**Published:** 2025-03-24

**Authors:** King Wa Tam, Dexing Zhang, Yiqi Li, Zijun Xu, Qiao Li, Yang Zhao, Lu Niu, Samuel YS Wong

**Affiliations:** 1 JC School of Public Health and Primary Care, The Chinese University of Hong Kong, Prince of Wales Hospital, Shatin, N.T., Hong Kong; 2 Cumming School of Medicine, University of Calgary, Calgary, Canada; 3 School of Nursing, The Hong Kong Polytechnic University, Hung Hom, KLN, Hong Kong; 4 The George Institute for Global Health, University of New South Wales, Kensington, Australia; 5 The George Institute for Global Health, Beijing, China; 6 Xiangya School of Public Health, Central South University, Changsha, China

**Keywords:** Meaning in life, Depression, Anxiety, Loneliness, Cohort, Primary care

## Abstract

**Background:**

Depression, anxiety and loneliness are common among older patients. As a potential psychological buffer against these challenges, meaning in life (MIL) remains underexplored in longitudinal studies within this population. This study aims to examine the longitudinal relationship of MIL with depression, anxiety, and loneliness among older adults with multimorbidity in Hong Kong.

**Methods:**

In a prospective cohort of 1077 primary care patients aged 60 or above with multimorbidity in Hong Kong, MIL was assessed using an item from the Chinese Purpose in Life test at baseline, the 1st follow-up (median: 1.3 years), and the 2nd follow-up (median: 3.1 years). Depression, anxiety, and loneliness were assessed using the Patient Health Questionnaire, Generalized Anxiety Disorder, and De Jong Gierveld Loneliness scales, respectively, at each time point. Cross-lagged relationships between MIL and these measures were examined using cross-lagged panel models.

**Results:**

Participants had an average age of 70.0 years, with 70.1% being female. Higher MIL predicted lower depression (β = -0.15), anxiety (β = -0.13), overall loneliness (β = -0.18), emotional loneliness (β = -0.15), and social loneliness (β = -0.16) at the 1st follow-up. Additionally, higher MIL predicted lower overall loneliness (β = -0.12), emotional loneliness (β = -0.11), and social loneliness (β = -0.10) at the 2nd follow-up. At baseline, higher depression (β = -0.21), overall loneliness (β = -0.15), emotional loneliness (β = -0.11), and social loneliness (β = -0.11), but not anxiety, predicted lower MIL at the 1st follow-up. At the 1st follow-up, depression (β = -0.23), anxiety (β = -0.16), overall loneliness (β = -0.10), and emotional loneliness (β = -0.11), but not social loneliness, predicted lower MIL at the 2nd follow-up.

**Conclusions:**

The findings suggest a bidirectional relationship between MIL and mental health outcomes in older patients with multimorbidity in Hong Kong. Emotional loneliness demonstrated a more consistent bidirectional association with MIL than social loneliness. Further research is needed to understand the underlying mechanisms and develop targeted interventions addressing both MIL and mental health problems.

**Supplementary Information:**

The online version contains supplementary material available at 10.1186/s12877-025-05762-7.

## Introduction

The global population of individuals aged 60 and above is projected to rise from 1 billion in 2019 to 2.1 billion by 2050 [1]. This trend is even more pronounced in Hong Kong, where the older adult population is expected to nearly double from 1.27 million to 2.44 million over the next two decades [2]. Mental health issues are common among older adults, as consistently shown in systematic reviews and meta-analyses. Across studies and regions, the prevalence of depression among older adults ranges from 7.7 to 81.1%, with an average of 31.74% [3]. The prevalence of anxiety disorders in the older population varies from 1.2 to 14% in community settings and from 1 to 28% in clinical settings [4]. Loneliness prevalence among older adults ranges from 5.9 to 43.3% [5].

Amid a global demographic shift towards an aging population, the concept of meaning in life (MIL) assumes a pivotal role in unravelling the intricate dynamics linking aging, health, and overall well-being [6]. MIL is a multifaceted construct encompassing three key dimensions: comprehension, which reflects an individual’s ability to understand life experiences; purpose, which pertains to the pursuit of meaningful goals and aspirations; and significance, the perception of one’s life as valuable and impactful [7]. Together, these dimensions create a sense of coherence, purpose, and worth in life. MIL evolves dynamically across life stages. In adulthood, it often derives from work, hobbies, and personal relationships, contributing to a sense of identity and purpose. Work or occupation serves as a significant source of MIL by providing purpose, achievement, and a sense of contribution to society. As individuals transition into old age, the focus shifts towards reflecting on life experiences and building a legacy. Additionally, MIL is profoundly influenced by cultural and religious worldviews, which shape how individuals interpret and construct meaning in their lives [8]. Importantly, research has shown that MIL is modifiable across different populations [9], indicating its potential as an effective prevention and treatment target for better well-being.

Previous research suggests that MIL plays a significant role in predicting overall well-being among older adults, particularly those dealing with chronic conditions. A strong sense of meaning is associated with better health status and, consequently, less healthcare utilization among older adults [10–12]. Its benefits for physical health are demonstrated by reduced risks for all-cause mortality [13–15], heart attacks [16], cardiovascular disease [14, 17, 18], stroke [18, 19], and sleep disturbances [20]. MIL is also a protective factor against suicide [21], cognitive decline [21, 22], and limitations in activities of daily living [16], contributing to psychosocial well-being. Furthermore, individuals with higher levels of MIL show higher resilience, represented by better emotional recovery from negative stimuli [23], greater pain tolerance [24], and higher acceptance of their chronic conditions [25]. In general, greater psychological well-being [11, 25–28] and better adjustment after disease diagnosis [29–31] have been observed in individuals with higher MIL in vast cross-sectional studies. Specifically, purpose in life has been negatively correlated with depression [11, 32, 33], anxiety [32–34], and loneliness [35]. A more comprehensive investigation of the causal direction and dynamics is provided by a few studies using a longitudinal design. One study showed that MIL appeared to offset the negative impact of traumatic life events on depressive symptoms during the same time period, but these stress-buffering effects were not significant when changes in depressive symptoms over time were examined [36]. Another longitudinal study with over 16,000 middle-aged and older adults in European countries found bidirectional, prospective associations of MIL with depression and loneliness [16].

Despite the growing body of research, there are still many research gaps regarding MIL and mental health. An important one is the prospective associations between MIL and health or quality of life outcomes, as extensive research has relied on cross-sectional data [10, 11, 26–32, 34, 35]. Only a handful of longitudinal studies have been conducted in this context, leaving the temporal relationships and directions of effects between MIL and mental health outcomes still poorly understood. Furthermore, existing longitudinal studies are marked by limitations such as small sample size [37], a single follow-up [16], and solely unidirectional effects of MIL on depression [27]. In addition, while there is extensive research on older adults in general, there is rarely a longitudinal study focusing specifically on older adults with multimorbidity. This focus is particularly important, as multimorbidity is common in older adults [38], and those with multimorbidity face additional burdens that increase their vulnerability to mental health problems [39–41]. By understanding prospective associations, primary care professionals can better enhance MIL and improve mental health outcomes for this specific group. In light of these research gaps, the current study stands as one of the first to use longitudinal data featuring more than two time points to analyse the bidirectional relationship between MIL and mental health in a Chinese older adult population with multimorbidity in primary care.

This study hypothesizes a bidirectional relationship between MIL and depression, anxiety, and loneliness based on several pathways proposed in the literature with some empirical evidence. *The first type* of pathway involves the consequences of self-regulatory processes that naturally arise from the affective, cognitive, and motivational components of MIL. The presence of meaning often elicits positive feelings, such as enjoyment [42, 43], satisfaction and fulfilment [44], and a sense of connectedness and belongingness [45, 46]. Positive affect can provide feedback regarding satisfactory progress towards one’s goals [47, 48], acting as a buffer against psychological distress. Moreover, MIL provides a cognitive framework that helps individuals perceive patterns, consistency, and significance in their environment [49–52], reducing negative or distorted thinking patterns that contribute to anxiety and depression. In addition, MIL motivates active engagement in behaviours that benefit mental health, such as health-promoting behaviours [50], initiating and maintaining social relationships [45, 46, 53–55], and engaging in prosocial behaviours [56], which are important to older adults with multimorbidity. The belief that life is worth caring for motivates individuals to do something good for themselves and others [45, 53, 57]. Finally, MIL facilitates more adaptive coping and resource mobilization to effectively solve problems and manage stressful life events [50, 52, 58–60], playing a crucial role in preventing and reducing anxiety and depression and in strengthening the ability to cope with loneliness [61]. *The second type* of pathway involves attentional and evaluative processes that shape MIL and may be affected by adverse mental health status. Acting as immediate prompts for appraisal, both affective states and belongingness can influence MIL by altering sensitivity to the relevance of an experience to MIL and its intensity [46, 62, 63]. Furthermore, anxiety, depression, and loneliness are often accompanied by negative cognitive biases that hinder individuals including older adults from finding and recognizing meaning in their lives, such as self-criticism, intrusive rumination, negative thinking, and distorted perception and evaluation of others [64–67]. Additionally, anxiety and depression reduce engagement in activities through which people can experience meaning in their lives, especially social interactions [68–73]. They may also divert individuals from choosing activities that align better with their values or prevent full immersion in activities, thus missing opportunities to enhance their MIL through positive experiences [68, 74–77]. Similarly, loneliness may make people focus more on social threats and rejection instead of long-term goals that are more important in finding the meaning of their lives [78, 79].

### Objectives

This study aimed to examine the longitudinal relationship of meaning in life (MIL) with depression, anxiety, and loneliness among primary care patients with multimorbidity in Hong Kong. We hypothesize that MIL will have a bidirectional relationship with depression, anxiety, and loneliness. Multimorbidity has previously been associated with depression in the Chinese population [80–82]. These findings can help in understanding whether MIL may serve as a buffer against the emotional burden caused by multimorbidity and assist in designing effective interventions for better well-being among older individuals with multimorbidity.

## Methods

### Study design

This prospective cohort study involved primary care patients with multimorbidity (cohort registration number ChiCTR-OIC-16008477). Details of the cohort have been previously published [83]. Ethics approval was obtained from The Joint Chinese University of Hong Kong (CUHK) - New Territories East Cluster (NTEC) Clinical Research Ethics Committee (CREC) (Reference No. CREC2016.204).

### Setting and participants

Participants were recruited from four public general outpatient clinics (GOPCs) in the New Territories East Cluster (NTEC) in Hong Kong. These government-funded general outpatient clinics managed by the Hospital Authority are located in a high-density residential region with a population of approximately 800,000 in 2018. Primary care patients aged 60 years or above, with two or more chronic diseases, and who could speak and understand Chinese were eligible for recruitment. Participants were initially screened at the GOPCs for eligibility and then invited for a scheduled face-to-face interview at a teaching clinic for detailed assessments.

At baseline, 1077 participants were recruited between 7 June 2016 and 23 October 2017. The 1st follow-up survey was conducted between 3 April 2018 and 6 March 2019 (*n* = 736), and the 2nd follow-up survey was conducted via telephone between 24 March and 15 April 2020 (*n* = 690). The median duration for the 1st follow-up was 484.5 days (IQR: 184.75), and for the 2nd follow-up, it was 1134.5 days (IQR: 258.5).

At baseline and the 1st follow-up, trained research staff conducted assessments face-to-face at the teaching clinic. For the 2nd follow-up assessment in 2020, trained research staff conducted a telephone survey for the convenience of patients due to the COVID-19 outbreak. The baseline survey consisted of questions on physical health, mental health, and demographic factors [83]. Sociodemographic information was collected at baseline, including age, sex, years of education, employment status, and marital status, and information on chronic disease diagnoses was obtained from the electronic clinical management records of the participating clinics.

### Measures

#### Meaning in life

Meaning in life was measured using a single item from the validated and reliable Chinese Purpose in Life test (CPIL), which comprises 20 items and five factors, with a Cronbach’s alpha of 0.84 and Guttman’s split-half reliability coefficient of 0.82 [84]. The item used is from the “Meaning of Existence” subscale and asks participants the question “My personal life is…” on a seven-point Likert scale from 1 (“Not meaningful and purposeful at all”) to 7 (“Very meaningful and purposeful”), with the midpoint 4 as the neutral response. This item has been used in other studies among older adults [12] and in other contexts [85–88]. It was introduced halfway through the baseline assessment of the cohort. In addition to the baseline assessment, the MIL measure was also assessed at the 1st and the 2nd follow-ups.

#### Depression

Depression was measured using the 9-item Patient Health Questionnaire (PHQ-9), a self-report questionnaire that assesses the presence and severity of depressive symptoms over the past two weeks. The PHQ-2 and PHQ-9 are valid and reliable tools for detecting depression in primary care [89], with the PHQ-2 designed to be used as an initial screening step [90]. The Chinese version of the PHQ-9 and the shorter PHQ-2 have been validated for the Chinese population [91–93]. Each item is rated on a 4-point scale from 0 (not at all) to 3 (nearly every day), with a total score ranging from 0 to 27. Scores between 5 and 9, 10–14, 15–19, and 20 + indicate mild, moderate, moderately severe, and severe depression, respectively. The PHQ-2 was used at baseline to screen for depression, and only those with a PHQ-2 score of at least 3 (*n* = 208) were asked to complete the PHQ-9, while the full PHQ-9 measure was administered to all participants at the 1st and 2nd follow-ups.

#### Anxiety

Anxiety was measured using the 7-item Generalized Anxiety Disorder (GAD-7) scale, a self-report questionnaire that assesses the presence and severity of anxiety symptoms over the past two weeks. The GAD-7 is a valid and reliable tool for detecting anxiety disorders in primary care. The shorter GAD-2 has been shown to retain the excellent psychometric properties of the GAD-7 [94] and has been proposed as a first step in screening for anxiety disorders [95]. Each item is rated on a 4-point scale from 0 (not at all) to 3 (nearly every day), with a total score ranging from 0 to 21. Scores between 5 and 9, 10–14, and 15–21 indicate mild, moderate, and severe anxiety, respectively. The GAD-2 was used at baseline to screen for anxiety [94], and only those with a GAD-2 score of at least 3 (*n* = 184) were asked to complete the GAD-7, while the full GAD-7 measure was administered to all participants at the 1st and 2nd follow-ups.

#### Loneliness

Loneliness was measured using the 6-item De Jong Gierveld Loneliness Scale (DJGLS) [96]. This scale has been validated for assessing loneliness in Chinese older adults [97]. It contains two subscales assessing social and emotional loneliness, as well as an overall loneliness score. Scores from 0 to 1 indicate little to no loneliness, scores from 2 to 4 indicate moderate loneliness, and scores from 5 to 6 indicate severe loneliness [98]. The DJGLS was assessed at baseline, the 1st follow-up, and the 2nd follow-up. A group of individuals (*n* = 285) did not complete the DJGLS at baseline because initially, only those with a PHQ-2 score of at least 3 were asked to complete it, as the aim was to measure loneliness levels among those who were depressed at the beginning. In the end, the DJGLS was administered to 792 participants at baseline, with 588 of them having a PHQ-2 score lower than 3.

### Statistical analysis

In this study, cross-lagged panel models were used to examine the relationship between meaning in life and another mental health variable of interest (i.e., depression, anxiety, or loneliness) over time. The cross-lagged panel model is a type of structural equation modelling (SEM) that allows testing the directionality of the relationship between two variables by examining the temporal precedence of one variable over the other.

To fit each cross-lagged panel model, the model was specified by defining the cross-lagged paths between the outcome variables across time. The model also included covariances between the residuals of the outcome variables at each time point, as well as variances for each outcome variable. While the main models did not include other covariates, supplementary files include results that were adjusted for age, sex, and the number of chronic diseases at baseline. The SEM was estimated using full information maximum likelihood via the lavaan function from the lavaan package in R, which can handle missing data [99]. The level of statistical significance was set at 5% (two-sided). Standardized coefficients are reported to allow direct comparisons of effect sizes.

Two goodness-of-fit measures were employed to assess the adequacy of model fit: the comparative fit index (CFI) and the standardized root mean square residual (SRMR). A CFI value above 0.95 indicates a good model fit [100], with values above 0.90 acceptable as a rule of thumb [101]. For the SRMR, values lower than 0.06 indicate a good fit, with acceptable values under 0.08 [100]. All data management and statistical analyses were performed using R version 4.3.0 [102].

## Results

### Participant characteristics

The demographic characteristics of the participants at baseline, the 1st follow-up and the 2nd follow-up are shown in Table 1. Among the 1077 participants at baseline, 341 and 387 were lost to the 1st and 2nd follow-ups, respectively. At baseline, the mean age of participants was 70.0 years (SD = 6.8), and 70.1% were female. The mean number of chronic diseases was 4.0 (SD = 1.8), and the mean number of years of education was 7.6 (SD = 4.3). The majority of participants were either retired (56.5%) or homemakers (35.4%) and were either married (67.3%) or widowed (23.1%). The response rates for T1 and T2 were 68.3% and 64.1% respectively. Those lost to follow-up were not statistically different in sociodemographic characteristics (*p* > 0.05) from respondents at the two time points, except for being marginally older by about 1 year.

**Table 1:** Descriptive statistics at baseline, 1^st^ follow-up, and 2^nd^ follow-up

	Baseline	T1	T2	*p*-value
**N**	1077	736	690	
**Age (Mean (SD))**	70.0 (6.8)	69.5 (6.1)	69.5 (6.1)	0.153
**Sex (N (%))**				0.599
**Female**	755 (70.1%)	520 (70.7%)	499 (72.3%)	
**Male**	322 (29.9%)	216 (29.3%)	191 (27.7%)	
**Number of chronic diseases** **(Mean (SD))**	4.0 (1.8)	4.0 (1.8)	4.0 (1.8)	0.915
**Years of education (Mean (SD))**	7.6 (4.3)	7.7 (4.2)	7.7 (4.2)	0.556
**Employment status (N (%))**				0.991
Retiree	608 (56.5%)	419 (56.9%)	387 (56.1%)	
Housemaker	381 (35.4%)	260 (35.3%)	250 (36.2%)	
Employee	74 (6.9%)	48 (6.5%)	45 (6.5%)	
Self-employed/employer	13 (1.2%)	9 (1.2%)	8 (1.2%)	
Not reported	1 (0.1%)	0 (0.0%)	0 (0.0%)	
**Marital Status (N (%))**				0.990
Married	725 (67.3%)	493 (67.0%)	457 (66.2%)	
Widowed	249 (23.1%)	170 (23.1%)	157 (22.8%)	
Divorced	52 (4.8%)	37 (5.0%)	40 (5.8%)	
Single	33 (3.1%)	25 (3.4%)	22 (3.2%)	
Separated	18 (1.7%)	11 (1.5%)	14 (2.0%)	

These variables were reported at baseline. N = number of observations. SD = standard deviation. The *p*-values indicate the level of significance of chi-squared tests on categorical/dichotomous variables and one-way ANOVA on numerical variables. Percentages (or standard deviations where specified) are in parentheses

The summary statistics of the outcome variables at baseline, the 1st follow-up, and the 2nd follow-up are shown in Table 2. The mean score for meaning in life was 4.85 (SD = 1.2) at baseline, 5.12 (SD = 1.27) at the 1st follow-up, and 5.25 (SD = 1.12) at the 2nd follow-up.

At baseline, the mean PHQ-2 score was 1.16 (SD = 1.49), and 18.9% of participants had a score of 3 or higher, indicating possible depression. Among those who screened positive and completed the PHQ-9 (*n* = 208), the mean score was 11.25 (SD = 4.42), and 58.2% (*n* = 121) had a score of 10 or higher, indicating moderate to severe depression. At the 1st follow-up, the mean PHQ-9 score was 4.21 (SD = 4.20), with 11.5% having moderate to severe depression (PHQ-9 ≥ 10). At the 2nd follow-up, the mean PHQ-9 score was 4.49 (SD = 4.41), with 11.0% having moderate to severe depression (PHQ-9 ≥ 10). Severe depression (PHQ-9 ≥ 20) was below 1% at follow-ups.

At baseline, the mean GAD-2 score was 1.21 (SD = 1.46), and 17.0% of participants had a score of 3 or higher, indicating possible anxiety. Among those who screened positive and completed the GAD-7 (*N* = 184), the mean score was 10.62 (SD = 3.94), and 58.7% had a score of 10 or higher, indicating moderate to severe anxiety. At the 1st follow-up, the mean GAD-7 score was 2.48 (SD = 3.91), with 6.5% having moderate to severe anxiety (GAD-7 ≥ 10). At the 2nd follow-up, the mean GAD-7 score was 2.96 (SD = 3.91), with 6.7% having moderate to severe anxiety (GAD-7 ≥ 10). Severe anxiety (GAD-7 score ≥ 15) was below 3% at follow-ups.

At baseline, the mean DJGLS score for loneliness was 1.76 (SD = 1.82), with 35.2% of participants experiencing moderate loneliness (DJGLS between 2 and 4) and 11.2% experiencing severe loneliness (DJGLS ≥ 5). The proportion of participants with severe loneliness increased considerably to 27.9% at the 2nd follow-up during the initial phase of COVID-19, driven by increases in both emotional and social loneliness.

**Table 2:** Summary statistics of outcome variables at baseline, 1^st^ follow-up, and 2^nd^ follow-up

	Baseline		T1		T2	
**Items**	N	N (%)/ Mean (SD)	N	N (%)/ Mean (SD)	N	N (%)/ Mean (SD)
**Meaning in life (1–7)**	544	4.85 (1.20)	736	5.12 (1.27)	687	5.25 (1.12)
**Depression (PHQ-2) (0–6)**	1077	1.16 (1.49)	736	0.98 (1.53)	690	1.31 (1.60)
< 3		873 (81.1%)		634 (86.1%)		560 (81.2%)
≥ 3		204 (18.9%)		102 (13.9%)		130 (18.8%)
**Depression (PHQ-9) (0–27)**	208	11.25 (4.42)	736	4.21 (4.20)	690	4.49 (4.41)
0–4		6 (2.9%)		475 (64.5%)		418 (60.6%)
5–9		81 (38.9%)		177 (24.0%)		196 (28.4%)
10–14		71 (34.1%)		63 (8.6%)		47 (6.8%)
15–19		36 (17.3%)		15 (2.0%)		25 (3.6%)
20+		14 (6.7%)		6 (0.8%)		4 (0.6%)
**Anxiety (GAD-2) (0–6)**	1077	1.21 (1.46)	736	0.82 (1.34)	690	1.06 (1.45)
< 3		894 (83.0%)		663 (90.1%)		609 (88.3%)
≥ 3		183 (17.0%)		73 (9.9%)		81 (11.7%)
**Anxiety (GAD-7) (0–21)**	184	10.62 (3.94)	736	2.48 (3.91)	688	2.96 (3.91)
0–4		9 (4.9%)		599 (81.4%)		504 (73.3%)
5–9		67 (36.4%)		89 (12.1%)		138 (20.1%)
10–14		83 (45.1%)		28 (3.8%)		30 (4.4%)
15+		25 (13.6%)		20 (2.7%)		16 (2.3%)
**Loneliness (DJGLS) (0–6)**	792	1.76 (1.82)	736	1.54 (1.77)	689	2.89 (2.00)
0–1		424 (53.5%)		444 (60.3%)		211 (30.6%)
2–4		279 (35.2%)		227 (30.8%)		286 (41.5%)
5–6		89 (11.2%)		65 (8.8%)		192 (27.9%)
**Emotional loneliness (0–3)**	792	0.95 (1.04)	736	0.73 (0.93)	689	1.22 (1.08)
**Social loneliness (0–3)**	792	0.81 (1.28)	736	0.82 (1.18)	689	1.66 (1.34)

N = number of observations. SD = standard deviation

### Relationship with depression

**Fig. 1:**
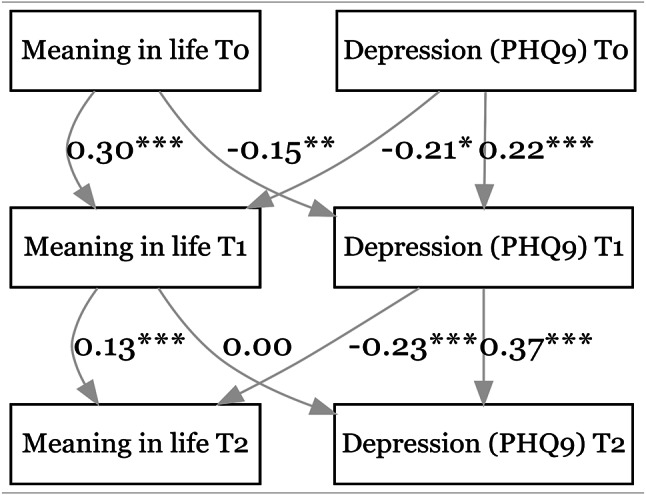
Relationship between meaning in life & depression (PHQ-9). CFI = 0.975. SRMR = 0.036. *p < 0.05, **p < 0.01, ***p < 0.001

The goodness-of-fit measures indicate a good model fit, with CFI between 0.948 and 0.975 and SRMR between 0.030 and 0.049 except for the model with social loneliness (CFI = 0.908, SRMR = 0.050). As shown in Fig. 1, higher MIL at an earlier time point significantly predicted lower levels of depression (PHQ-9) at the 1st follow-up (β = -0.15, *p* < 0.01), but not at the 2nd follow-up (β = 0.00, *p* = 0.89). Conversely, higher PHQ-9 scores at an earlier time point predicted lower MIL at both the 1st follow-up (β = -0.21, *p* = 0.02) and the 2nd follow-up (β = -0.23, *p* < 0.001). The effect of MIL at baseline on depression at the 1st follow-up becomes statistically insignificant when using the PHQ-2 instead of the PHQ-9 (see Figure S1 in the supplementary file).

### Relationship with anxiety

**Fig. 2:**
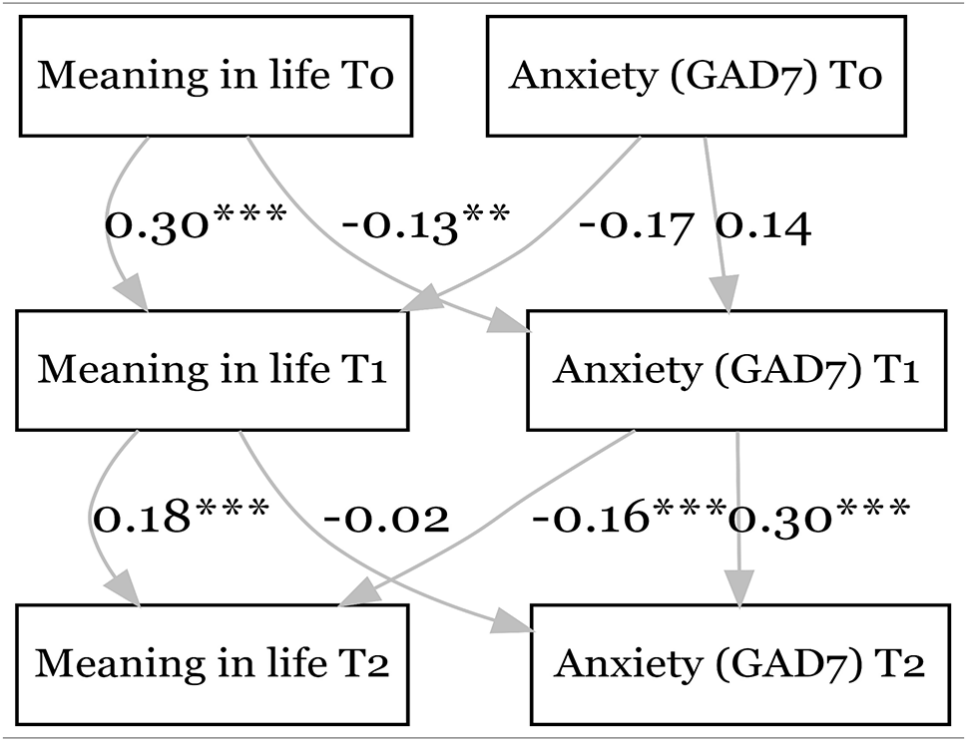
Relationship between meaning in life & anxiety (GAD-7). CFI = 0.974. SRMR = 0.030. *p < 0.05, **p < 0.01, ***p < 0.001

As shown in Fig. 2, higher MIL at an earlier time point was linked to lower levels of anxiety (GAD-7) at the 1st follow-up (β = -0.13, *p* < 0.01) but not at the 2nd follow-up (β = -0.02, *p* = 0.57). Conversely, higher GAD-7 scores at an earlier time point only predicted lower MIL at the 2nd follow-up (β = -0.16, *p* < 0.001) but not at the 1st follow-up (β = -0.17, *p* = 0.09). The effect of MIL at baseline on anxiety at the 1st follow-up remained statistically significant when using the GAD-2 instead of the GAD-7 (see Figure S2 in the supplementary file).

### Relationship with loneliness

**Fig. 3:**
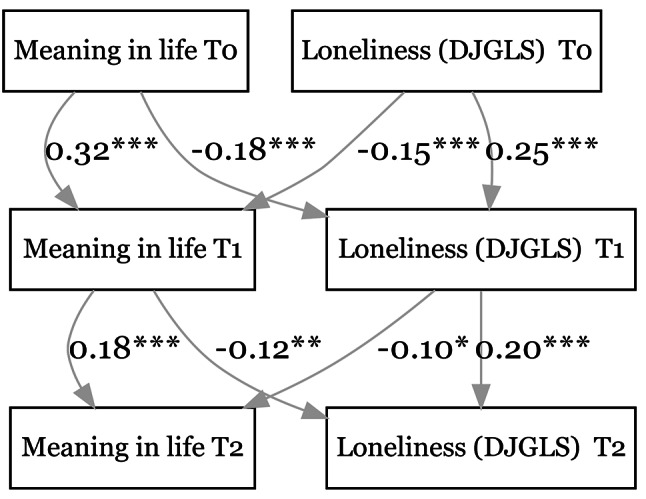
Relationship between meaning in life & loneliness (DJGLS). CFI = 0.949. SRMR = 0.049. *p < 0.05, **p < 0.01, ***p < 0.001

Figure 3 shows that higher MIL at an earlier time point significantly predicted lower levels of loneliness (DJGLS) at both the 1st follow-up (β = -0.18, *p* < 0.001) and the 2nd follow-up (β = -0.12, *p* < 0.01). Similarly, higher DJGLS scores at an earlier time point predicted lower MIL at both the 1st follow-up (β = -0.15, *p* < 0.001) and the 2nd follow-up (β = -0.10, *p* = 0.01).

**Fig. 4:**
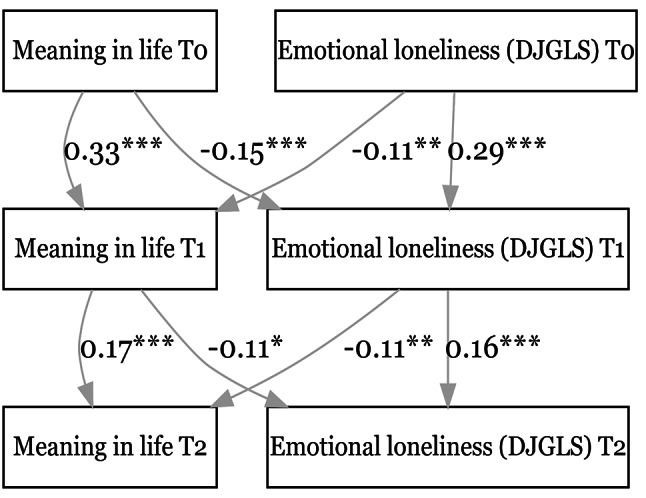
Relationship between meaning in life & emotional loneliness (DJGLS subscale). CFI = 0.964. SRMR = 0.037. *p < 0.05, **p < 0.01, ***p < 0.001

Figure 4 shows that higher MIL at an earlier time point predicted lower levels of emotional loneliness (DJGLS emotional subscale) at both the 1st follow-up (β = -0.15, *p* < 0.001) and the 2nd follow-up (β = -0.11, *p* = 0.01). Likewise, higher emotional loneliness scores at an earlier time point predicted lower MIL at both the 1st follow-up (β = -0.11, *p* < 0.01) and the 2nd follow-up (β = -0.11, *p* < 0.01).

**Fig. 5:**
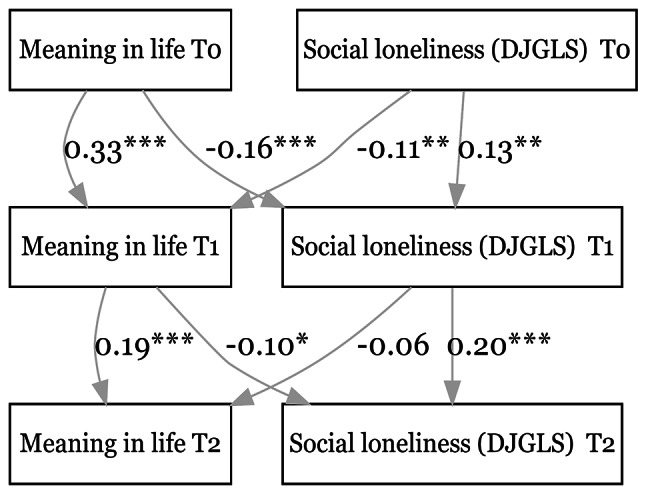
Relationship between meaning in life & social loneliness (DJGLS subscale). CFI = 0.908. SRMR = 0.050. *p < 0.05, **p < 0.01, ***p < 0.001

Furthermore, Fig. 5 shows that higher MIL at an earlier time point predicted lower levels of social loneliness (DJGLS social subscale) at both the 1st follow-up (β = -0.16, *p* < 0.001) and the 2nd follow-up (β = -0.10, *p* = 0.02). However, higher social loneliness scores at an earlier time point were associated with lower MIL at the 1st follow-up (β = -0.11, *p* < 0.01) but not at the 2nd follow-up (β = -0.06, *p* = 0.13). These results also show that the outcome variables predict themselves at follow-ups. The only exception is anxiety as measured by GAD-7 due to a lack of samples at baseline, but anxiety at baseline, as measured by GAD-2 with the full sample, also predicts itself at the first follow-up (Figure S2 in the supplementary file). Moreover, these results do not meaningfully change when further adjusted for age, sex, and the number of chronic diseases at baseline (Figures S3 to S9 in the supplementary file).

## Discussion

This study examined the longitudinal relationship between MIL and mental health outcomes, including depression, anxiety, and loneliness, among primary care patients with multimorbidity in Hong Kong. The results showed that higher MIL predicted lower levels of depression, anxiety, and loneliness at the 1st follow-up and lower levels of loneliness at the 2nd follow-up. Higher levels of depression and loneliness predicted lower MIL at both the 1st and 2nd follow-ups, whereas only anxiety at the 1st follow-up predicted lower MIL at the 2nd follow-up. MIL consistently predicted lower levels of emotional and social loneliness at both time points. Likewise, emotional loneliness predicted lower MIL at both time points, while social loneliness predicted lower MIL only at the 1st follow-up but not at the 2nd follow-up. While previous studies from Hong Kong identified a negative correlation between MIL and depression [103, 104], our study indicates that this relationship may be predominantly characterized as depression influencing MIL rather than vice versa. A 2023 meta-analysis found a stronger negative correlation between MIL and mental health issues, particularly depression, in Asian countries (Pearson correlation coefficient, − 0.48) than in Western countries (− 0.34), though the difference was not statistically significant [105]. Recent research also indicates that MIL plays a crucial role in mediating the effects of loneliness on depressive symptoms among older Korean adults during the COVID-19 pandemic [106]. Similarly, another recent study across Europe and Israel has shown that MIL mediated over 80% of the association between loneliness and depression [107]. Overall, these findings suggest a bidirectional relationship between MIL and depression, anxiety, and loneliness among older patients with multimorbidity in Hong Kong.

The above findings are consistent with previous studies that have found a reciprocal association between MIL and mental health outcomes among middle-aged and older adults [16, 37]. For instance, using different instruments, prospective bidirectional associations were found between MIL and depression and loneliness over a 6-year follow-up in a cohort of over 16,000 middle-aged and older adults from 13 European countries [16]. Despite a smaller sample size and shorter study period, the present study observed reciprocal relationships at more time points, revealing similar patterns among a cohort with multimorbidity. In addition, this study found that emotional loneliness played a more prominent role than social loneliness in predicting MIL.

Notably, some of the bidirectional relationships observed in this study did not remain consistent over time. While MIL showed statistically significant prospective influences on depression and anxiety in the 1st follow-up period, these prospective effects were no longer statistically significant in the 2nd follow-up period. On the other hand, the prospective influence of anxiety on MIL was statistically insignificant in the 1st follow-up period but became statistically significant in the 2nd follow-up period. This inconsistency could result from two possible scenarios — methodological issues or instability of the true associations.

In the first scenario, the true associations are stable over time, but this study failed to detect them due to methodological issues, including changes in the data collection method, measurement error, and potential confounders. First, due to the outbreak of COVID-19, telephone interviews replaced in-person interviews, and the 2nd follow-up period became somewhat longer (with a median interval 31% longer than the 1st follow-up). These changes may have introduced biases and obscured the effects, particularly due to the change in interview mode [108]. Second, the influence of measurement error was unclear and may depend on the specific instrument used. For example, when employing PHQ-2 to measure depression instead of PHQ-9, the statistically significant prospective associations between MIL and depression in the 1st follow-up period became statistically insignificant. In contrast, replacing GAD-7 with GAD-2 to measure anxiety did not affect the statistical significance of the prospective associations between MIL and anxiety. This may indicate that the more comprehensive PHQ-9 scale with higher specificity [89] captures the relationship of depression with MIL better than the screening-oriented PHQ-2, whereas GAD-2 did not appear to substantially attenuate the power of detecting the relationship of anxiety with MIL compared to GAD-7. Third, time-dependent confounders that emerged after the 1st follow-up may have concealed the effects of MIL. Such confounders include life events that are common at older ages and cause overwhelming distress, such as the loss of a significant other, changes in socioeconomic status, and the development of chronic conditions. Older adults generally report a greater sense of purpose and meaning in life, while younger individuals often search for meaning [6, 16]. The sense of purpose in life declines in late adulthood with age acting as a proxy for significant life events such as illness, retirement, and widowhood [16]. However, this progression may not be strictly linear or monotonic; for instance, purpose in life can continue to increase in the oldest old aged 90–105 who reported greater purpose in life than those aged 80–89 [109]. Maintaining high levels of meaningfulness in old age might become more challenging due to age-related losses, such as health issues, retirement, and widowhood.

In the second scenario, the observed inconsistency over time may not be a result of methodological issues but rather reflect instability in the true associations. Such instability could arise from natural developments or external factors. First, the findings may suggest that the protective effects of MIL on mental health outcomes attenuate at a certain age. This aligns with a five-year follow-up study of individuals older than 85, where no predictive or protective link between purpose in life and depression was identified [110]. Second, a major event during the 2nd follow-up period, namely the COVID-19 outbreak, profoundly impacted people’s lives in many aspects, possibly altering the reciprocal relationships between MIL and mental health outcomes. The onset of the COVID-19 pandemic adversely affected the psychosocial health of this cohort, as previously reported [111]. A recent study found that Chinese individuals with greater MIL experienced more stress and anxiety from increased media consumption when bored during the early phase of the COVID-19 outbreak in late January 2020 [112]. Overall, our results suggest that the relationship between MIL and depression or anxiety may have been more susceptible to the impacts of the COVID-19 outbreak, whereas the bidirectional relationship between MIL and loneliness appeared to be less affected. This could be because the extraordinary stressors caused by the pandemic may have overwhelmed older adults, exceeding what individuals could manage through their sense of meaning, thereby disrupting that specific relationship. In contrast, due to the nature of loneliness, individuals may have turned to their sense of meaning as a coping mechanism during times of crisis to navigate feelings of isolation.

In addition to the findings on bidirectional relationships and their changes over time, a new contribution of this study is that emotional loneliness has a stronger and more consistent relationship with MIL than social loneliness. This may reflect the different dimensions of loneliness captured by the DJGLS subscales. Emotional loneliness refers to the absence of close emotional attachments or intimate relationships, while social loneliness refers to the lack of a broader network of social contacts or friends [97]. Although not encompassed within the DJGLS framework, existential loneliness has been identified as a dimension of loneliness distinct from emotional and social loneliness. It reflects a lack of meaning in life and disconnection from a greater purpose, experienced as an intrinsic sense of separateness that cannot be easily remedied through social interactions or relationships [113, 114]. In other words, meaning in life and existential loneliness are interconnected concepts. As recent research suggests that the three dimensions of loneliness are correlated [113], this might explain the more consistent bidirectional relationship between meaning in life and loneliness compared to depression and anxiety. Emotional loneliness may have a greater impact on MIL because it is associated with a lack of meaningful social relationships [113], while social loneliness may be more influenced by situational factors such as the availability of social resources and opportunities. Similarly, it is worth investigating the extent to which existential loneliness is connected to emotional loneliness, as this could explain its stronger bidirectional relationship with MIL compared to social loneliness. Moreover, emotional loneliness may be more challenging to alleviate than social loneliness, as it requires more than simply increasing the quantity or frequency of social interactions. Therefore, interventions targeting emotional loneliness and existential loneliness may be more effective in enhancing MIL than those addressing only social loneliness. Examples of interventions for emotional loneliness include support groups, cognitive behavioural therapy, and emotion-focused therapy [115, 116]. Interventions for existential loneliness include logotherapy, meaning-centred or existential interventions, mindfulness and acceptance-based interventions, and philosophical counselling [117–120].

### Strengths and limitations

This study has several strengths. First, this is one of the first studies with more than one follow-up to examine the bidirectional relationship between MIL and mental health outcomes among older patients with multimorbidity. Second, it used validated measures of MIL and mental health outcomes that have been widely used in previous research. It also has a relatively large sample size and a long follow-up period which increased the statistical power and generalizability of the findings.

However, this study also has a few limitations in addition to the issues already discussed above that could explain the somewhat inconsistent bidirectional relationships (i.e., data collection method, potential confounders, and COVID-19). First, only a single-item measure of MIL was used, which may not capture the full complexity and multidimensionality of the construct. Second, it may be subject to bias from self-report measures and loss to follow-up. Third, a low number of participants who screened positive for depression or anxiety were measured using the full scales at baseline, which may have introduced selection bias and reduced the representativeness of the sample, even though missing data were accounted for using the full information maximum likelihood method. Fourth, as this study only included an older population from Hong Kong, the relationships between MIL and mental health among younger populations should be considered in future studies.

### Implications for services and future research

These findings have important implications for services that aim to improve the psychological well-being of older patients with multimorbidity. Services should consider incorporating interventions that enhance MIL among this population, such as meaning-centred psychotherapy [121] and mindfulness [122], along with activities that can facilitate positive mood, self-esteem, autonomy, competence, social connections, and religiosity/spirituality [8]. “Restorying” interventions may also be valuable for increasing MIL, as they help individuals reframe their personal experiences and life stories according to the narrative of an archetypal hero’s journey [123]. Enhancing the presence of MIL may be more effective for improving mental health outcomes in older adults. A 2020 meta-analysis showed that the presence of MIL positively correlates with subjective well-being, whereas the search for meaning often shows a weaker or negative association [124]. Services should also address the negative impact of depression, anxiety, and loneliness on MIL by providing appropriate treatment and support for these conditions. Moreover, services should pay attention to the different types of loneliness that may affect MIL and tailor interventions accordingly. For example, services may provide opportunities for emotional intimacy and attachment for those who suffer from emotional loneliness such as mental health support and establishing peer support networks. Alternatively, they could facilitate social participation and engagement for those who suffer from social loneliness such as through community and intergenerational programs.

Future research should employ a more comprehensive and multidimensional measure of MIL that can capture its different facets, including comprehension, purpose, and significance. Future research should also investigate how and to what extent the COVID-19 pandemic played a role in diminishing the predictive power of MIL on depression and anxiety, as the disruption may have occurred by blocking one or more of the mediating paths mentioned in the last section or caused additional distress that exceeded the capacity of MIL to cope as a psychological resource. Further studies are needed to understand the exact mechanisms, as well as other potential mediators or moderators that may explain or modify the effects of MIL on mental health outcomes, or vice versa, such as positive emotions, optimism, social support, or resilience.

## Conclusions

This study investigated the bidirectional relationship between meaning in life (MIL) and mental health outcomes such as depression, anxiety, and loneliness over time in older primary care patients with multimorbidity in Hong Kong. The results showed that MIL and mental health outcomes influenced each other reciprocally, with higher MIL leading to lower levels of depression, anxiety, and loneliness, and vice versa. The psychological burden experienced by older adults may be amplified by multimorbidity, making MIL an even more critical factor in mitigating anxiety, depression, and loneliness in this population. On the other hand, in a healthier population, the relationship between MIL and mental health might be less pronounced, as chronic health issues would not contribute as significantly to psychological distress. Future studies may look more closely at whether there are any differences in these relationships in healthy populations. The results also showed that emotional loneliness exhibited a more consistent bidirectional association with MIL than social loneliness. These findings suggest that MIL is a psychological resource that could act as a buffer against the negative impact of multimorbidity on mental health and that interventions enhancing MIL may improve the psychological well-being of older patients with multimorbidity. Therefore, healthcare professionals should consider referring patients with low MIL to psychologists or mental health specialists. A multidisciplinary approach that integrates insights from psychology, sociology, social work, health sciences, religious studies, and philosophy could lead to more effective interventions targeting both MIL and mental health issues. However, further research is necessary to elucidate the causal mechanisms and moderating factors of this relationship and to develop effective, tailored interventions targeting both MIL and mental health problems.

## Electronic supplementary material

Below is the link to the electronic supplementary material.

Supplementary Material 1

## Data Availability

No datasets were generated or analysed during the current study.
