# Supplementary material for: Meaning in life: bidirectional relationship with depression, anxiety, and loneliness in a longitudinal cohort of older primary care patients with multimorbidity
Source: BMC Geriatr. 2025 Mar 24;25:195. doi: 10.1186/s12877-025-05762-7 (PMC11931759; doi:10.1186/s12877-025-05762-7)
Supplement: Supplementary file 1 — Supplementary Material 1 [file 12877_2025_5762_MOESM1_ESM.docx]

## Supplementary file


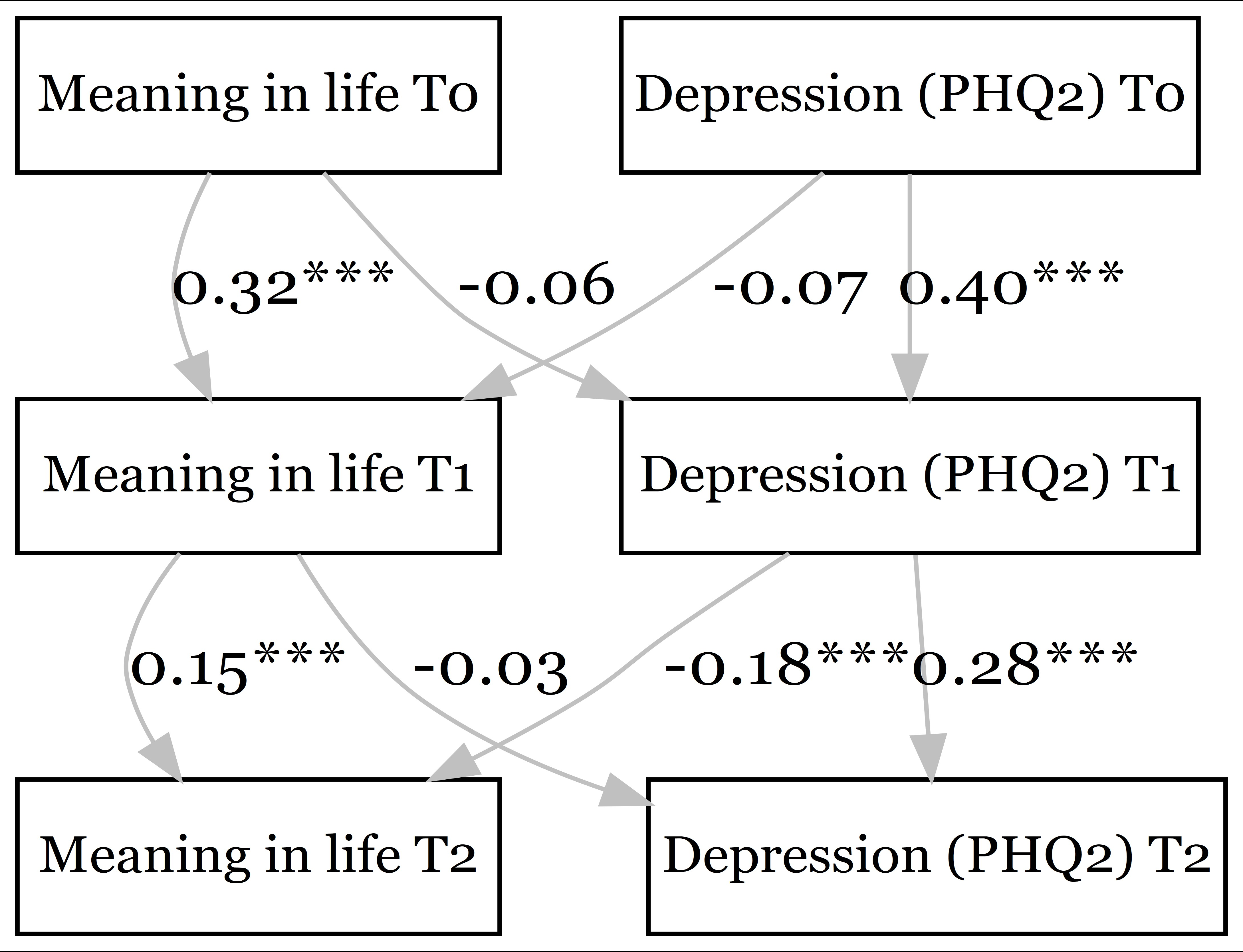


Figure S1. Relationship between meaning in life & depression (PHQ-2)

CFI = 0.959. SRMR = 0.032. *p < 0.05, **p < 0.01, ***p < 0.001.


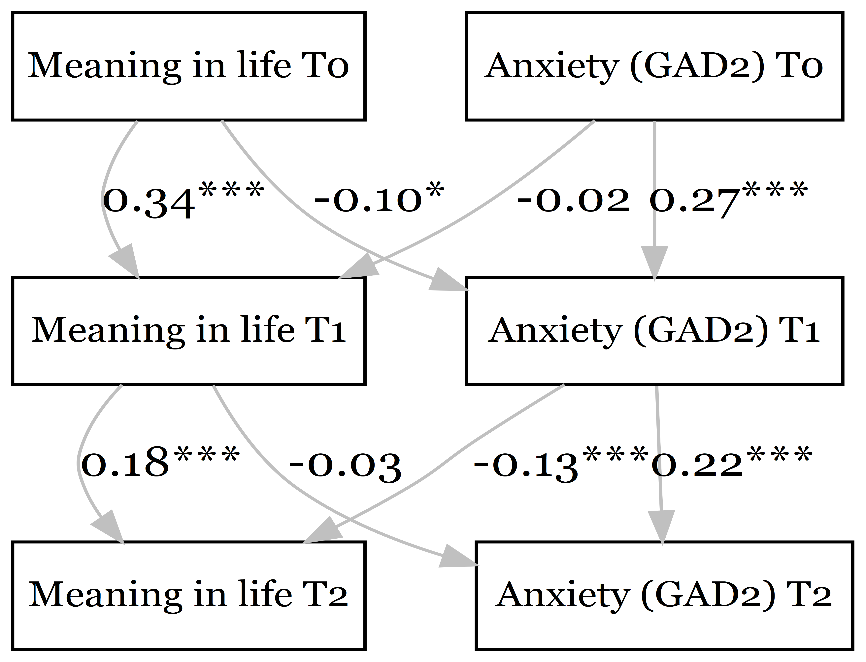


Figure S2. Relationship between meaning in life & anxiety (GAD-2)

CFI = 0.948. SRMR = 0.033. *p < 0.05, **p < 0.01, ***p < 0.001.


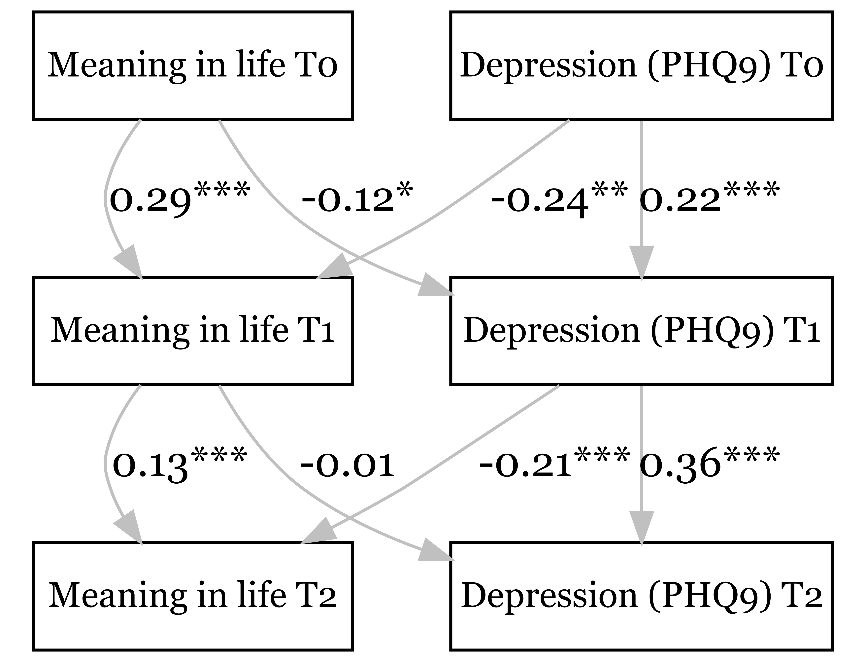


Figure S3. Relationship between meaning in life & depression (PHQ-9), adjusted for age, sex and number of chronic diseases at baseline

CFI = 0.978. SRMR = 0.025. *p < 0.05, **p < 0.01, ***p < 0.001.


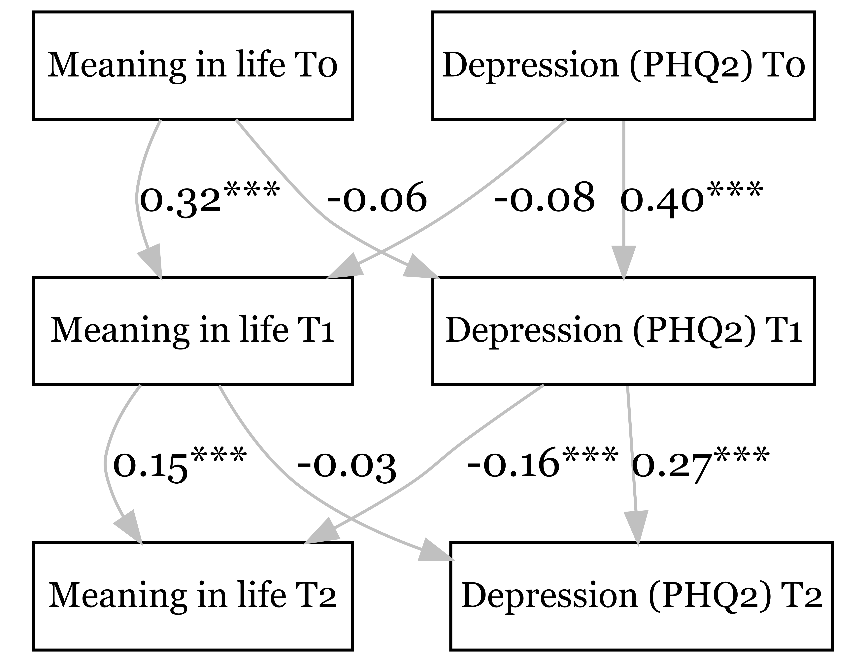


Figure S4. Relationship between meaning in life & depression (PHQ-2), adjusted for age, sex and number of chronic diseases at baseline

CFI = 0.976. SRMR = 0.018. *p < 0.05, **p < 0.01, ***p < 0.001.


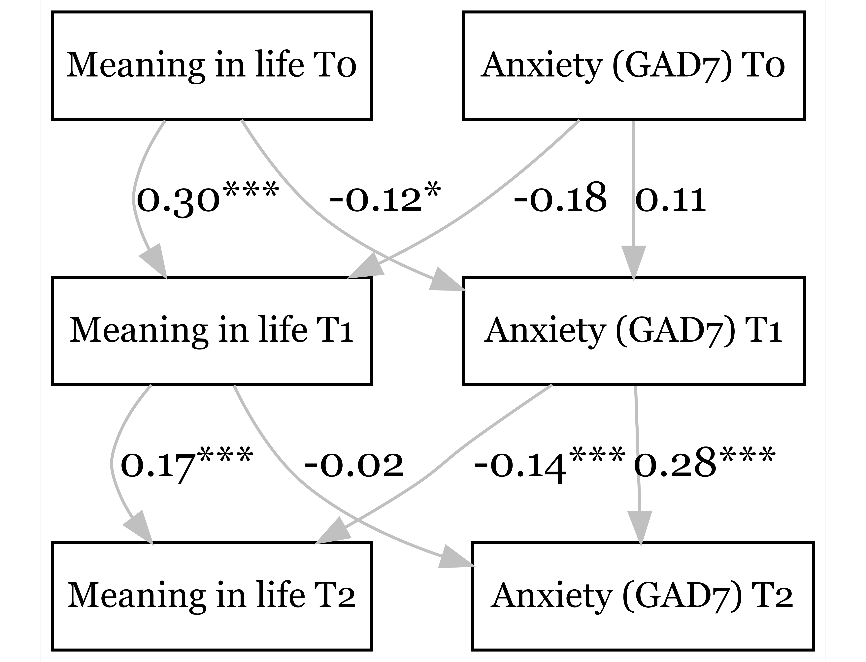


Figure S5. Relationship between meaning in life & anxiety (GAD-7), adjusted for age, sex and number of chronic diseases at baseline

CFI = 0.986. SRMR = 0.019. *p < 0.05, **p < 0.01, ***p < 0.001.


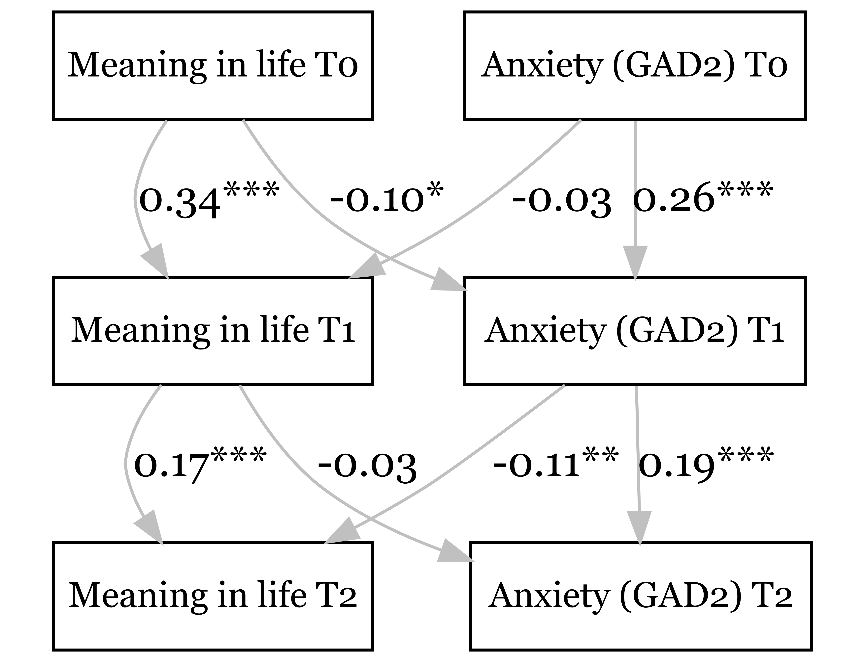


Figure S6. Relationship between meaning in life & anxiety (GAD-2), adjusted for age, sex and number of chronic diseases at baseline

CFI = 0.980. SRMR = 0.018. *p < 0.05, **p < 0.01, ***p < 0.001.


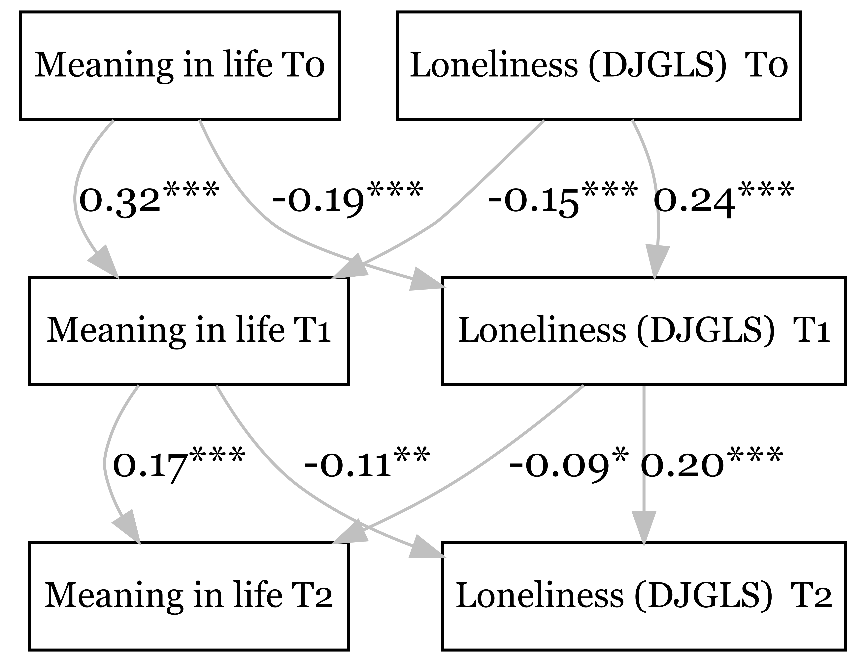


Figure S7. Relationship between meaning in life & loneliness (DJGLS), adjusted for age, sex and number of chronic diseases at baseline

CFI = 0.964. SRMR = 0.031. *p < 0.05, **p < 0.01, ***p < 0.001.


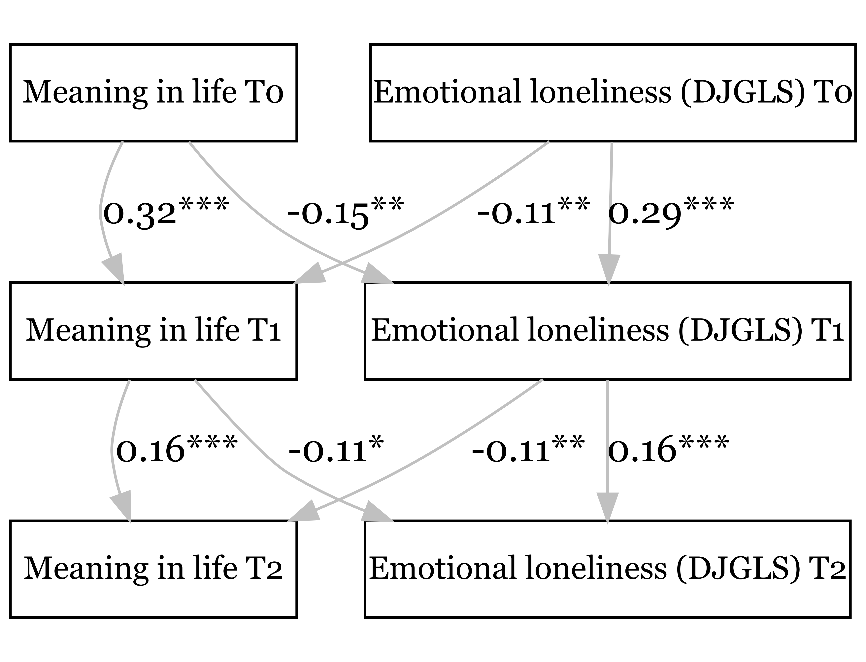


Figure S8. Relationship between meaning in life & emotional loneliness (DJGLS subscale), adjusted for age, sex and number of chronic diseases at baseline

CFI = 0.975. SRMR = 0.023. *p < 0.05, **p < 0.01, ***p < 0.001.


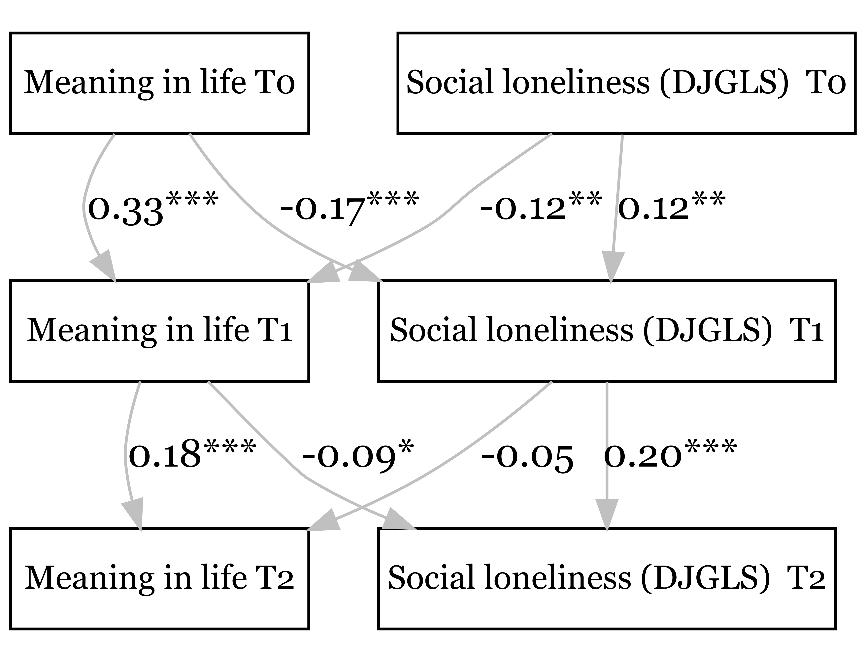


Figure S9. Relationship between meaning in life & social loneliness (DJGLS subscale), adjusted for age, sex and number of chronic diseases at baseline

CFI = 0.935. SRMR = 0.032. *p < 0.05, **p < 0.01, ***p < 0.001.
